# Intestinal epithelial injury and inflammation after physical work in temperate and hot environments in older men with hypertension or type 2 diabetes

**DOI:** 10.1113/EP092567

**Published:** 2025-03-30

**Authors:** Ben J. Lee, Tessa R. Flood, Sophie L. Russell, James J. McCormick, Kelli E. King, Naoto Fujii, Tatsuro Amano, Sean Notley, Glen P. Kenny

**Affiliations:** ^1^ Occupational and Environmental Physiology Group, Centre for Physical Activity, Sport and Exercise Sciences Coventry University Coventry UK; ^2^ Institute of Sport Manchester Metropolitan University Manchester UK; ^3^ Human and Environmental Physiology Research Unit, School of Human Kinetics University of Ottawa Ottawa Quebec Canada; ^4^ Faculty of Health and Sport Sciences University of Tsukuba Tsukuba Japan; ^5^ Laboratory for Exercise and Environmental Physiology, Faculty of Education Niigata University Niigata Japan

**Keywords:** ageing, cytokines, heat stress, hypertension, intestinal fatty acid binding protein, lipopolysaccharide‐binding protein, plasma soluble cluster of differentiation 14, type 2 diabetes

## Abstract

We tested whether older adults with well‐controlled type 2 diabetes or hypertension, compared with age‐matched adults without chronic disease, exhibit greater intestinal damage, microbial translocation and inflammation during exertional heat stress. Twelve healthy men (age 59 years, SD 4 years), nine with type 2 diabetes (age 60 years, SD 5 years) and nine with hypertension (age 60 years, SD 4 years) walked for 180 min at 200 W/m^2^ in temperate conditions (wet‐bulb globe temperature 16°C) and high‐heat stress conditions (wet‐bulb globe temperature 32°C). Serum intestinal fatty acid binding protein (IFABP), plasma soluble cluster of differentiation 14, lipopolysaccharide‐binding protein (LBP), interleukin‐6 and tumour necrosis factor‐alpha were measured pre‐ and postexercise and after 60 min recovery. Total exercise duration was lower in men with hypertension and diabetes (*p* ≤ 0.049), but core temperature did not differ. All markers increased more in heat versus temperate conditions (*p *< 0.002). In the heat, individuals with type 2 diabetes had greater postexercise increases in IFABP [+545 pg/mL (95% confidence interval: 222, 869)] and LBP [+3.64 µg/mL (1.73, 5.56)] relative to healthy control subjects (*p *< 0.048), but these resolved after recovery. Despite reduced exercise duration, hypertensive individuals showed similar increases in IFABP and LBP to control subjects. Our findings suggest that older workers with well‐controlled type 2 diabetes or hypertension might have greater vulnerability to heat‐induced gastrointestinal barrier disturbance and downstream inflammatory responses when compared with otherwise healthy, age‐matched adults during prolonged exercise in the heat.

## Abstract (highlights)

- **What is the central question of this study?**
  Do older men with well‐controlled type 2 diabetes or hypertension exhibit greater intestinal enterocyte damage, indicators of microbial translocation and inflammation during occupationally relevant exertional heat stress when compared with age‐matched men?
- **What is the main finding and its importance?**
  Heat stress increased intestinal damage and inflammatory responses in all groups, with type 2 diabetics showing greater vulnerability. Hypertensives had reduced exercise tolerance but similar systemic protein increases to control subjects, suggesting greater sensitivity to lower thermal doses. These findings highlight heat‐related vulnerability in older adults with well‐controlled health conditions.

## 1 INTRODUCTION

A globally ageing population alongside growing rates of chronic non‐communicable diseases will be reflected in labour force demographics, with the number of workers aged >50 years expected to grow rapidly (United States Census Bureau, 2024). Many physically demanding occupations are increasingly being performed in extreme heat, placing workers at an elevated risk of heat illnesses and mortality (Flouris et al., 2022). Older adults aged ≥65 years exposed to heat waves are particularly vulnerable to heat injury, which is characterized by hyperthermia (i.e., increased core temperature) and evidence of end‐organ damage (e.g., kidneys, gastrointestinal tract and liver) in the absence of heat stroke (Meade et al., 2020). Older workers will continue to carry the burden of excess illnesses and mortality from extremes of heat (Ioannou et al., 2022), primarily owing to age‐related decrements in heat dissipation, which are exasperated by the presence of chronic non‐communicable diseases, such as type 2 diabetes and hypertension (Kenny et al., 2013; Kenny, Sigal et al., 2016; Notley et al., 2021; Yardley et al., 2013). Despite our growing understanding of the physiological consequences of prolonged work in the heat in heat‐vulnerable workers (i.e., older adults with and without chronic disease) (De Barros et al., 2022b; Lee et al., 2023, 2024; Meade et al., 2020; Notley et al., 2021), there remains a paucity of information regarding the cellular and molecular mechanisms that might underlie greater vulnerability to heat injuries in older adults both with and without chronic diseases (Chapman & Schlader, 2025).

Exercise in hot environments confers direct (thermal) and indirect (splanchnic vasoconstriction) stress on the gastrointestinal mucosa (Rowell, 1974). Decreased gut perfusion contributes to epithelial injury, an acute localized inflammatory reaction and alterations in the phosphorylation status of endothelial tight junctions, leading to increased gastrointestinal permeability and the translocation of lipopolysaccharide (LPS) into the systemic circulation (Zuhl et al., 2014). There, LPS binds Toll‐like receptor‐4 on surveilling leucocytes, activating the nuclear factor kappa B cascade and resulting in the production of pro‐inflammatory cytokines (Kuennen et al., 2011). Overactivation of this pathway (e.g., systemic inflammatory response syndrome) confers the greater core temperature rise, intravascular coagulation and multiple organ failure that characterize exertional heat stroke (Chin & Mackinnon, 2006). Ageing is accompanied by the gradual deterioration of the gastrointestinal barrier, which has been closely linked to the progressive deterioration of systemic health and the gradual appearance of metabolic defects (Conway et al., 2024; Wilson et al., 2018). Increased intestinal barrier permeability has also been identified as a risk factor for certain metabolic diseases, such as type 2 diabetes (Cox et al., 2017). Therefore, it is plausible that ageing workers with and without chronic illness are at a greater risk of exertional heat injury secondary to gastrointestinal barrier dysfunction. In support of this, we have recently shown that serum intestinal fatty acid binding protein (IFABP), a marker of endothelial cell damage, was ∼308 pg/mL greater in healthy older relative to young adults after prolonged (∼180 min), moderate‐intensity exercise (∼200 W) in hot (∼41°C, 35% relative humidity), but not temperate (∼22°C, 35% relative humidity) conditions (Lee et al., 2024). We observed no differences in two acute phase proteins that are indirect surrogate indicators of microbial translocation [soluble cluster of differentiation 14 (sCD14) and lipopolysaccharide‐binding protein (LBP)] in either cohort, suggesting that the enterocyte damage was neither severe nor prolonged enough to initiate substantial translocation of microbial products (Bennett et al., 2020). Whether this response might be mediated in older adults with common chronic diseases associated with gastrointestinal barrier dysfunction (Gomes et al., 2017; Yuan et al., 2021; Zhou et al., 2016), chronic low‐grade inflammation (Gomes et al., 2017) and metabolic endotoxaemia (Cox et al., 2017) remains unclear.

In this exploratory study, we sought to examine changes in a surrogate marker of enterocyte damage (IFABP), two acute phase proteins that provide an indirect assessment of microbial translocation (CD14 and LBP) and systemic markers of inflammation [interleukin‐6 (IL‐6) and tumour necrosis factor‐alpha (TNFα)] in healthy older men and in older men with type 2 diabetes or hypertension, before and after a 3 h period of moderate‐intensity work (∼200 W/m^2^), as defined by the American Conference of Governmental Industrial Hygienists action limit values and threshold limit values, in both temperate and hot environments. Given our prior observations of an age‐related increase in enterocyte damage following exercise in the heat, but not in temperate conditions, we tested the hypothesis that enterocyte damage, surrogate markers of immune activation, lipopolysaccharide exposure and inflammation would be increased in men with well‐controlled type 2 diabetes and hypertension relative to older counterparts without disease following exercise in hot ambient conditions.

## 2 MATERIALS AND METHODS

### 2.1 Ethical approval

The experiment was approved by the University of Ottawa Health Sciences and Research Ethics Board (H04‐17‐05), and experiments were carried out in accordance with the latest revision of the *Declaration of Helsinki*, except for registration in a database. Written and informed consent was obtained from all volunteers before their participation.

### 2.2 Participants

The present study was part of a larger investigation evaluating heat strain in young and older adults, the procedures of which are described in detail elsewhere (Notley et al., 2021). Blood samples were collected from 30 healthy and habitually active (engaging in physical activity on ≥3 days per week) older adults [aged 59 years (range: 52–65 years), *n* = 12] and older adults with either well‐controlled type 2 diabetes [aged 60 years (range: 50–65 years), *n* = 9] or well‐controlled hypertension [aged 60 years (range: 55–65 years), *n* = 9]. Prospective participants were eligible if they were 65–85 years old, non‐smoking, spoke English or French, and were able to provide informed consent (both males and females were eligible). Exclusion criteria included physical restriction (e.g., owing to disease: intermittent claudication, renal impairment, active proliferative retinopathy, unstable cardiac or pulmonary disease, disabling stroke, severe arthritis), use of or changes in medication judged by the patient or investigators to make participation in this study inadvisable (e.g., medications increasing the risk of heat‐related illness; β‐blockers, anticholinergics), cardiac abnormalities identified via 12‐lead ECG during an incremental exercise test to volitional fatigue (performed for all participants). Type 2 diabetics were included if they had been diagnosed ≥5 years before inclusion in the study (mean ± SD; 10 ± 5 years) and had a haemoglobin A1C between 6% and 10% (mean ± SD; 7.0% ± 0.6%). Participants with hypertension were included if they had been diagnosed ≥5 years prior (mean ± SD: 9 ± 4 years) and/or had an average systolic blood pressure of >140 mmHg or diastolic blood pressure of >90 mmHg, measured using an automated sphygmomanometer, taking the average of the lower two of three readings. All participants were on prescribed medications for their conditions, which they adhered to throughout the study period, with their conditions considered ‘well controlled’ by their medical practitioner. Participant characteristics are presented in Table 1.

**TABLE 1:** Participant characteristics for each experimental group.

Parameter	Healthy controls	Type 2 diabetics	Hypertensives
*n*	12	9	9
Age, years	59 (52–65)	60 (50–65)	60 (55–65)
Body mass index, kg/m^2^	28 (23–34)	29 (24–39)	28 (24–31)
Body mass, kg	83 (69–104)	84 (66–117)	84 (72–99)
Body fat percentage	24 (14–35)	26 (20–34)	28 (24–31)
Body surface area, m^2^	2.0 (0.1)	2.0 (0.1)	2.0 (0.1)
Resting blood pressure, mmHg			
Systolic	124 (115–135)	123 (111–134)	129 (115–143)
Diastolic	77 (71–84)	75 (67–87)	80 (71–86)
Mean	93 (87–101)	91 (83–102)	96 (88–103)
Physical activity, min/week	291 (150–480)	259 (75–540)	198 (60–300)^a^
Peak O_2_ uptake, mL/kg/min	39.5 (30.5–51.5)	30.2 (21.5–43.5)^a^	33.7 (26.0–44.9)
Haemoglobin A1C	–	7.0 (6.0–7.8)	–

*Note*: Data are presented as the group mean and the range. Of the nine individuals with controlled type 2 diabetes, two were taking six medications, one was taking five medications, one was taking four medications, two were taking three medications, two were taking two medications, and one was taking one medication. Classes of medication consisted of metformin (*n* = 9), insulin (*n* = 3), diuretics (*n* = 1), angiotensin‐converting enzyme inhibitors (*n* = 2), angiotensin receptor blockers (*n* = 1), calcium channel blockers (*n* = 1), sulphonylureas (*n* = 2), incretin mimetics (*n* = 1), Sodium‐glucose cotransporter‐2 inhibitors (*n* = 2), Dipeptidyl peptidase‐4 inhibitor (*n* = 2) and statins (*n* = 7). Of the nine individuals with controlled hypertension, one was taking three medications, five were taking two medications, two were taking one medication, and one was on no medication. Classes of medication consisted of statins (*n* = 4), calcium channel blockers (*n *= 3), angiotensin‐converting enzyme blockers (*n* = 4), angiotensin receptor blockers (*n* = 3) and diuretics (*n* = 1).

^a^ Different from older (*p *< 0.05).

Participants completed one screening session and two experimental sessions. Each trial was completed at the same time of day (within participants), and all trials were separated by >48 h. All participants were familiarized with the experimental procedures during the screening session and were provided with standardized test instructions before each experimental visit. During the screening session, the height and body mass of participants were assessed and used to calculate body surface area (Du Bois & Du Bois, 1989). Maximal aerobic capacity was determined using an incremental cycling protocol performed on a semi‐recumbent cycle ergometer (Corival; Lode BV, Groningen, The Netherlands) completed in thermoneutral conditions (∼23°C).

### 2.3 Pretrial standardization

For all study visits, participants arrived after consuming their habitual breakfast and were requested to refrain from vigorous exercise and to abstain from alcohol, caffeine and anti‐inflammatory drugs for a minimum of 24 h before participation in each experimental condition. Adherence was verified via a short questionnaire on arrival for each experimental visit. Each trial was completed at the same time of day (within participants), and all trials were separated by >48 h. Participants were instructed to consume ∼200–500 mL of water 2 h before their arrival to the laboratory, which was confirmed upon arrival by a urine specific gravity of <1.025.

### 2.4 Experimental trials

After confirming euhydration (urine specific gravity of <1.025) and nude body mass (IND 560; Mettler Toledo Inc., Mississauga, ON, Canada), participants self‐inserted a rectal temperature probe (Mallinckrodt Medical, St Louis, MO, USA) 12 cm past the anal sphincter. Participants were then instrumented with a heart rate monitor (Polar M400 monitor; Polar Electro, Finland) in a temperate room (23°C). After ∼30 min, baseline heart rate and rectal temperature were recorded. Participants then donned a single‐layer T‐shirt, single‐layer cotton coveralls (100% cotton), socks and sports shoes (total insulation ∼1 Clo). Participants entered a climate‐controlled chamber with minimal airflow (< 0.3 m/s) regulated to 35% relative humidity and one of two air temperatures (21.9°C or 41.4°C), equating to a wet‐bulb globe temperature of 16°C (temperate) or 32°C (hot). After 30 min of seated habituation, participants were asked to walk for 180 min on a treadmill at a metabolic rate of 200 W/m^2^, followed by 60 min of seated recovery within the chamber. Water maintained at the respective chamber temperature was available ad libitum. Trials were terminated upon the request of the participant or if core body temperature exceeded 39.5°C.

### 2.5 Blood collection

Venous blood samples were collected in either serum separator tubes or EDTA‐coated tubes via venipuncture (antecubital) at the following time points: (1) during the final 5 min of baseline measurements; (2) after being seated for ≥10 min to account for fluid redistribution, immediately postexercise; and (3) during the final 5 min of the seated recovery period. Plasma samples were centrifuged (1380*g* relative centrifugal force) immediately for 10 min, whereas serum samples were left to clot before centrifugation. Plasma and serum were aliquoted and subsequently stored at −80°C until analysis.

Serum concentrations of IFABP and plasma concentrations of sCD14 and LBP were measured in duplicate using an enzyme‐linked immunosorbent assay (ELISA). Samples were diluted 1:2 (for IL‐6 and TNFα), 1:5 (for IFABP) or 1:800 (for sCD14 and LBP) in PBS and 1% bovine serum albumin before analysis. Owing to the well‐established difficulties in obtaining reliable systemic measurements of LPS concentrations (Ogden et al., 2020), in addition to limitations to commercially available LPS assays, microbial translocation was determined by circulating concentrations of LBP and sCD14, which are commonly used surrogates for LPS (Schumann, 1992). Optimal sample dilutions were determined from in‐house linearity and spike‐recovery experiments using the desired sample matrices (plasma or serum) and were performed before sample analysis. All remaining assay steps were conducted according to standard sandwich ELISA protocols (product codes: IFABP, DY3078; LBP, DY870; sCD14, DY383; IL‐6, S6050B; TNFα, DY210; Bio‐Techne, Oakville, ON, Canada). Absorbance for all assays was read on a plate reader (BMG SpectroStar Nano) at a wavelength of 450 nm. A wavelength correction (570 nm) was applied to minimize the effects of non‐specific wavelength emissions from microplate materials. The within‐ and between‐plate coefficients of variation were respectively as follows: IFABP (2.7% and 3.9%); sCD14 (4.2% and 6.0%); LBP (3.8% and 5.4%); IL‐6 (3.5% and 4.3%); and TNFα (1.8% and 2.3%). The percentage change in estimated plasma volume was calculated according to the equations of Dill and Costill (1974), incorporating the percentage change in albumin, which has been shown to have good correlation and agreement with traditional haematocrit‐ and haemoglobin‐derived estimations (*r* = 0.972; Alis et al., 2015). Statistical analysis was performed on the corrected data.

### 2.6 Statistical analysis

All analyses were performed using R (R Core Team, 2013). The homogeneity and normality of residuals were evaluated visually using residual and Q–Q plots before analysis. Data were analysed using mixed linear models. Initial models incorporated ‘temperature’ as a fixed effect to confirm a difference between the temperate and hot conditions. Thereafter, simplified models incorporating fixed effects for the group (healthy older, *n *= 12; type 2 diabetes, *n* = 9; and hypertension, *n *= 9) and time (baseline, end‐exercise and recovery) were conducted on data obtained from the hot condition. This approach was taken to minimize the number of comparisons made within each statistical model that were not aligned with the experimental hypothesis. Participant identity was modelled as a random effect (intercept), and exercise duration (in minutes) was incorporated as a covariate to account for differences in exercise tolerance time. In the event of a significant interaction or main effect, pairwise comparisons were performed using Student's unpaired (group) or paired [time (rest vs. exercise, rest vs. recovery)] two‐tailed *t*‐tests. To correct for multiple comparisons, the Bonferroni procedure was used. Within‐group differences are described as the mean (SD), and between‐group comparisons are described as the mean difference (95% confidence interval: lower, upper).

## 3 RESULTS

### 3.1 Exercise tolerance and thermoregulation

All healthy older adults and 17 of 18 older adults with type 2 diabetes or hypertension completed exercise in the temperate condition. In contrast, 5 of 12 older adults and 16 of 18 older adults with type 2 diabetes or hypertension terminated exercise prematurely in the hot condition. Exercise duration was lower in older adults with type 2 diabetes or hypertension [median (range): 115 min (60–180)] in comparison to older men without these conditions [170 min (78–180); *p *< 0.05]. There were no between‐group differences for end‐exercise or recovery body core temperature following exercise in temperate or hot conditions. For context, in the temperate condition peak end‐exercise body core temperature was 37.53°C (SD 0.29) in the healthy older group, 37.72°C (SD 0.37) in the older adults with hypertension and 37.86°C (SD 0.16) in the older adults with type 2 diabetes. In contrast, in the hot condition peak body core temperature was 38.88°C (SD 0.53) in the healthy older group, 38.71°C (SD 0.53) in the older adults with hypertension and 38.81°C (SD 0.37) in the older adults with type 2 diabetes. Table 2 summarizes key physiological responses to each experimental condition.

**TABLE 2:** Exercise tolerance time, body core temperature, mean skin temperature, heart rate and heart rate reserve for each experimental group at baseline and immediately at the end of prolonged moderate‐intensity exercise (200 W/m^2^) in temperate and hot conditions.

	Temperate (16°C WBGT)			Hot (32°C WBGT)		
	Older	Type 2 diabetes	Hypertension	Older	Type 2 diabetes	Hypertension
Exercise time, min	180 (0)	180 (0)	172 (24)	159 (31)	123 (42)^b^	117 (45)^[Link]^, ^[Link]^
*T*_rectal_, °C						
Baseline	36.9 (0.2)	37.1 (0.3)	37.1 (0.2)	37.1 (0.2)	37.2 (0.3)	37.1 (0.2)
End exercise	37.5 (0.3)	37.9 (0.4)	37.8 (0.4)	39.0 (0.3)^b^	38.8 (0.4)^b^	38.8 (0.4)^b^
*T*_skin_,°C						
Baseline	31.7 (0.6)	31.9 (0.7)	31.8 (0.5)	34.7 (0.4)^b^	34.8 (0.5)^b^	34.9 (0.4)^b^
End exercise	32.3 (0.8)	32.6 (0.7)	32.4 (1.1)	36.4 (0.7)^b^	36.4 (0.4)^b^	36.5 (0.6)^b^
Heart rate, beats/min						
Baseline	63 (10)	70 (11)	69 (14)	69 (10)	75 (10)	74 (12)
End exercise	97 (13)	107 (20)	113 (20)	137 (13)^b^	138 (21)^b^	142 (18)^b^
HRR, %						
End exercise	35 (9)	42 (13)	46 (16)	76 (12)^b^	81 (16)^b^	79 (17)^b^
HR_max_, % Trial average End exercise	58 (8) 61 (8)	65 (9) 67 (10)	59 (14) 64 (12)	68 (13) 85 (7)	73 (18) 89 (10)	67 (15) 83 (14)
Relative exercise intensity, % ${\dot{V}}_{O_{2}\mathrm{peak}}$	32 (10)	43 (11)	37 (7)	33 (10)	43 (10)^a^	38 (8)
Fluid consumption, L/h	1.09 (0.66)	0.84 (0.45)	0.76 (0.27)	2.19 (0.75)^b^	1.32 (0.33)^[Link]^, ^[Link]^	1.51 (0.50)^[Link]^, ^[Link]^
WBSR, kg/h/m^2^	0.18 (0.12)	0.15 (0.1)	0.23 (0.17)	0.32 (0.14)^b^	0.32 (0.17)^b^	0.39 (0.17)^b^
Body mass change, %	−0.10 (1.39)	−0.59 (0.40)	−0.39 (0.48)	−1.46 (1.28)^b^	−1.29 (1.39)^b^	−1.11 (1.18)^b^
Plasma volume change, %	−2.8 (5.6)	−1.0 (7.9)	0.6 (5.0)	−6.8 (6.0)^b^	−8.7 (11.3)^b^	−5.7 (3.1)^b^

*Note*: Data are presented as the mean (SD).

Abbreviations: HR_max_, maximum heart rate during the final 10 min of the trial; HRR, heart rate reserve; *T*_rectal_, rectal temperature; *T*_skin_, mean skin temperature; ${\dot{V}}_{O_{2}\mathrm{peak}}$, peak O_2_ uptake; WBGT, wet‐bulb globe temperature; WBSR, whole‐body sweat rate.

^a^Different from older (*p *< 0.05).

^b^Different from temperate (*p *< 0.05).

### 3.2 Intestinal epithelial injury and microbial translocation

There was a main effect of temperature for IFABP (*F* = 108.7, *p *< 0.0001), sCD14 (*F* = 8.8, *p *= 0.004) and LBP (*F* = 48.6, *p *< 0.0001), with temperature × time interactions for each protein (all *p *< 0.002), confirming that environmental temperature mediates the responses of these proteins. Separate mixed linear models were therefore used to analyse absolute protein responses to the temperate and hot conditions.

**FIGURE 1:**
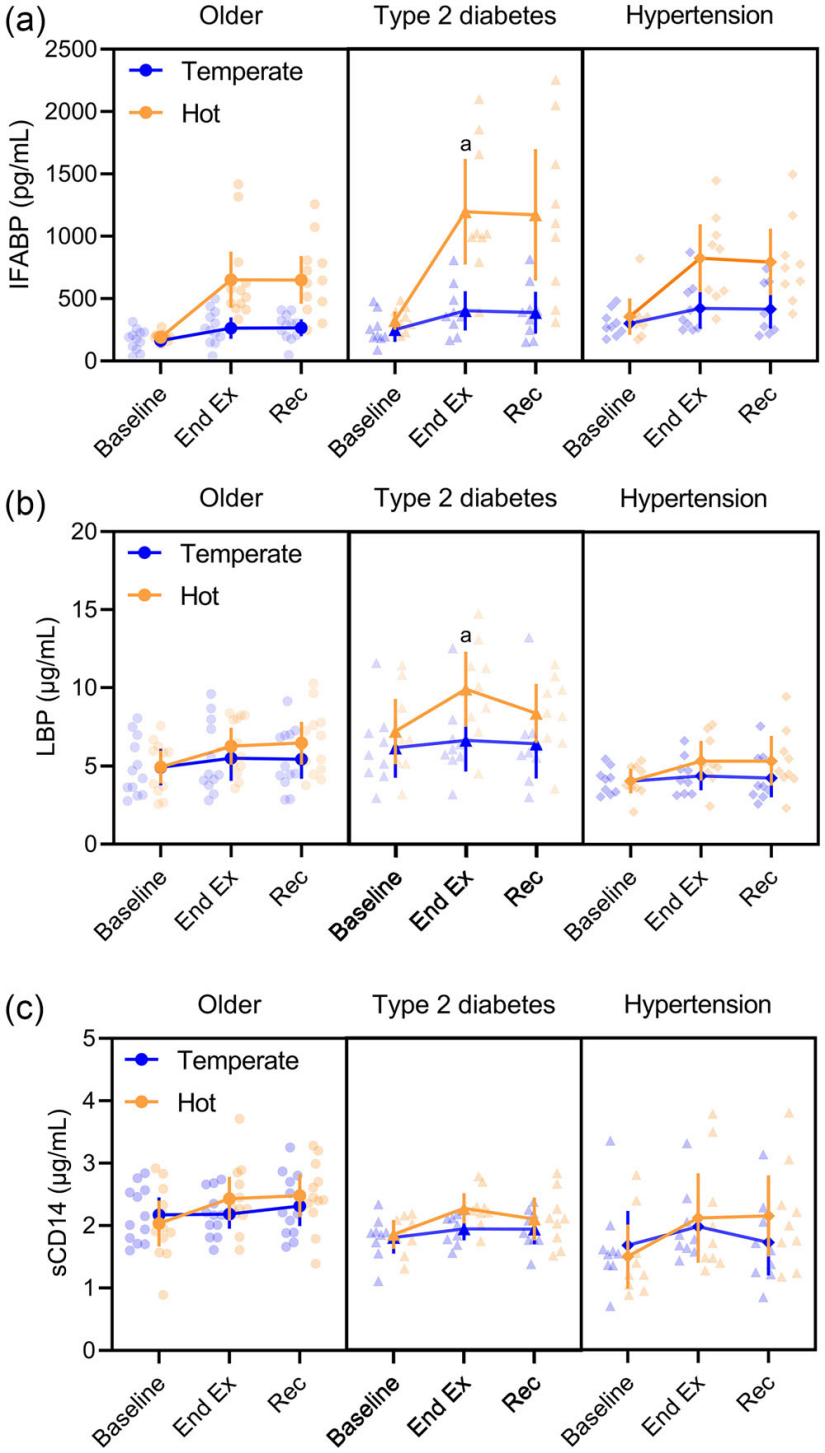
Absolute concentrations for markers of intestinal enterocyte damage and surrogate markers of microbial translocation during the temperate (blue) and hot (yellow) trials in healthy age‐matched men (*n* = 12; circles), men with type 2 diabetes (*n* = 9; triangles) and men with hypertension (*n* = 9; diamonds). Serum concentrations of intestinal fatty acid binding protein (IFABP; a), lipopolysaccharide binding protein (LBP; b) and soluble cluster of differentiation 14 (sCD14; c) are shown before (Baseline) and after exercise (End Ex) and following 60 min of recovery (Rec). Summary data are presented as the mean and SD. Individual data points are shown. Data were compared using mixed‐effects models, with main effects for time and group. ^a^Different from the healthy older group (*p *< 0.05).

In the temperate condition, a main effect for time was observed for IFABP (*F* = 22.4, *p *< 0.0001) and LBP (*F* = 4.3, *p *= 0.019) but not for sCD14 (*F* = 2.9, *p *= 0.07). There were no main effects for the group (all *p *> 0.07), nor a group × time interaction for IFABP, LBP or sCD14 (all *p *> 0.17; Figure 1). IFABP increased from baseline by 124 pg/mL (SD 133; *p *< 0.0001) at the end of the exercise, remaining elevated by 118 pg/mL (SD 124; *p *< 0.00001) after recovery. LBP increased from baseline by 0.46 µg/mL (SD 0.29; *p *= 0.019, Bonferroni adjusted) but was no different from baseline concentrations following recovery (*p *= 0.15).

In the hot condition, the main effects of time (all *p *< 0.0001), group (all *p *< 0.002) and a group × time interaction (all *p *< 0.047) were observed for IFABP and LBP, and a main effect of time (*p *< 0.0001) was observed for sCD14. Following exercise, IFABP increased by 462 pg/mL (SD 317) in the old group, 872 pg/mL (SD 621) in the older adults with type 2 diabetes, and 470 pg/mL (SD 232) in the older adults with hypertension (all *p *< 0.0001). IFABP remained elevated in all groups after recovery (all *p *< 0.001). Relative to the older group, IFABP was 545 pg/mL (222, 869) greater in the older adults with type 2 diabetes at the end of exercise (*p *= 0.048), but it was not different between groups following recovery [mean difference 522 pg/mL (199, 846); *p *= 0.07). There were no differences in IFABP between older adults without and older adults with hypertension at any time point.

**FIGURE 2:**
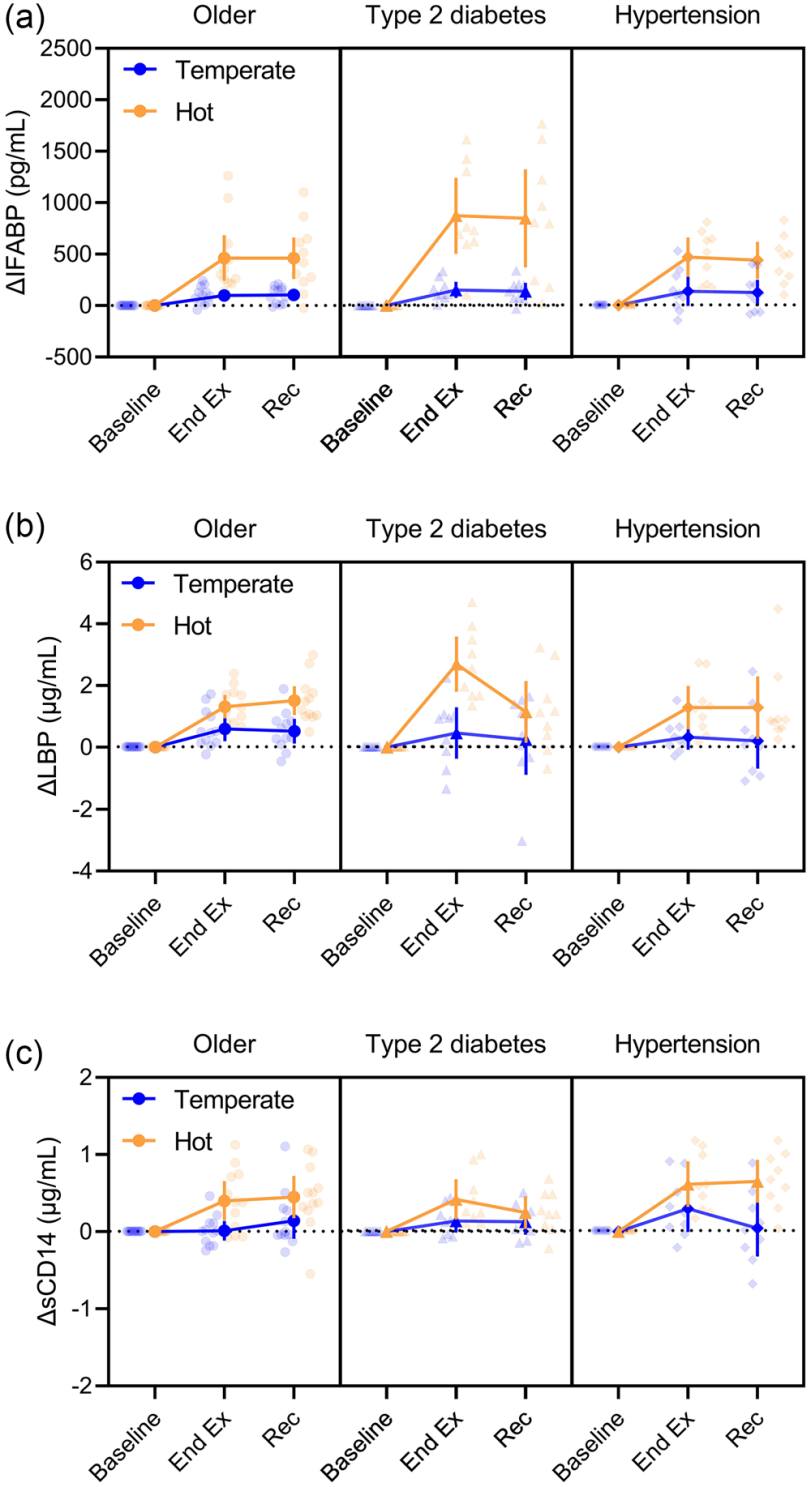
The change in concentrations from baseline for markers of intestinal enterocyte damage and surrogate markers of microbial translocation during the temperate (blue) and hot (yellow) trials in healthy age‐matched men (*n* = 12; circles), men with type 2 diabetes (*n* = 9; triangles) and men with hypertension (*n* = 9; diamonds). Serum concentrations of intestinal fatty acid binding protein (IFABP; a), lipopolysaccharide binding protein (LBP; b) and soluble cluster of differentiation 14 (sCD14; c) are shown before (Baseline) and after exercise (End Ex) and following 60 min of recovery (Rec). Summary data are presented as the mean and SD. Individual data points are shown.

LBP increased by 1.32 µg/mL (SD 0.59) in the old group, by 2.70 µg/mL (SD 1.16) in older adults with type 2 diabetes, and by 1.28 µg/mL (SD 0.92) in older adults with hypertension (all *p *< 0.001) after exercise and remained elevated in all groups following recovery (all *p *< 0.001). Relative to the older group, LBP was 3.64 µg/mL (1.73, 5.56) greater in the older adults with type 2 diabetes compared at the end of exercise (*p *= 0.017), and not different between groups following recovery [mean difference 1.90 µg/mL (0.01, 3.81), *p *= 1.00]. There were no differences in LBP between the older adults without and older adults with hypertension at any time point. sCD14 increased by 0.48 µg/mL (SD 0.38) following exercise and remained elevated by 0.45 µg/mL (SD 0.39) following recovery (both *p *< 0.001). The changes in IFABP, LBP and sCD14 from baseline in both the temperate and hot conditions are presented in Figure 2.

### 3.3 Systemic inflammatory responses

There was a main effect of temperature for IL‐6 (*F* = 34.3, *p *< 0.001), but not for TNFα (*F* = 2.3, *p *= 0.13), although there was a temperature × time interaction for both cytokines (both *p *< 0.01). Separate mixed linear models were therefore used to analyse IL‐6 and TNFα responses to the temperate and hot conditions.

**FIGURE 3:**
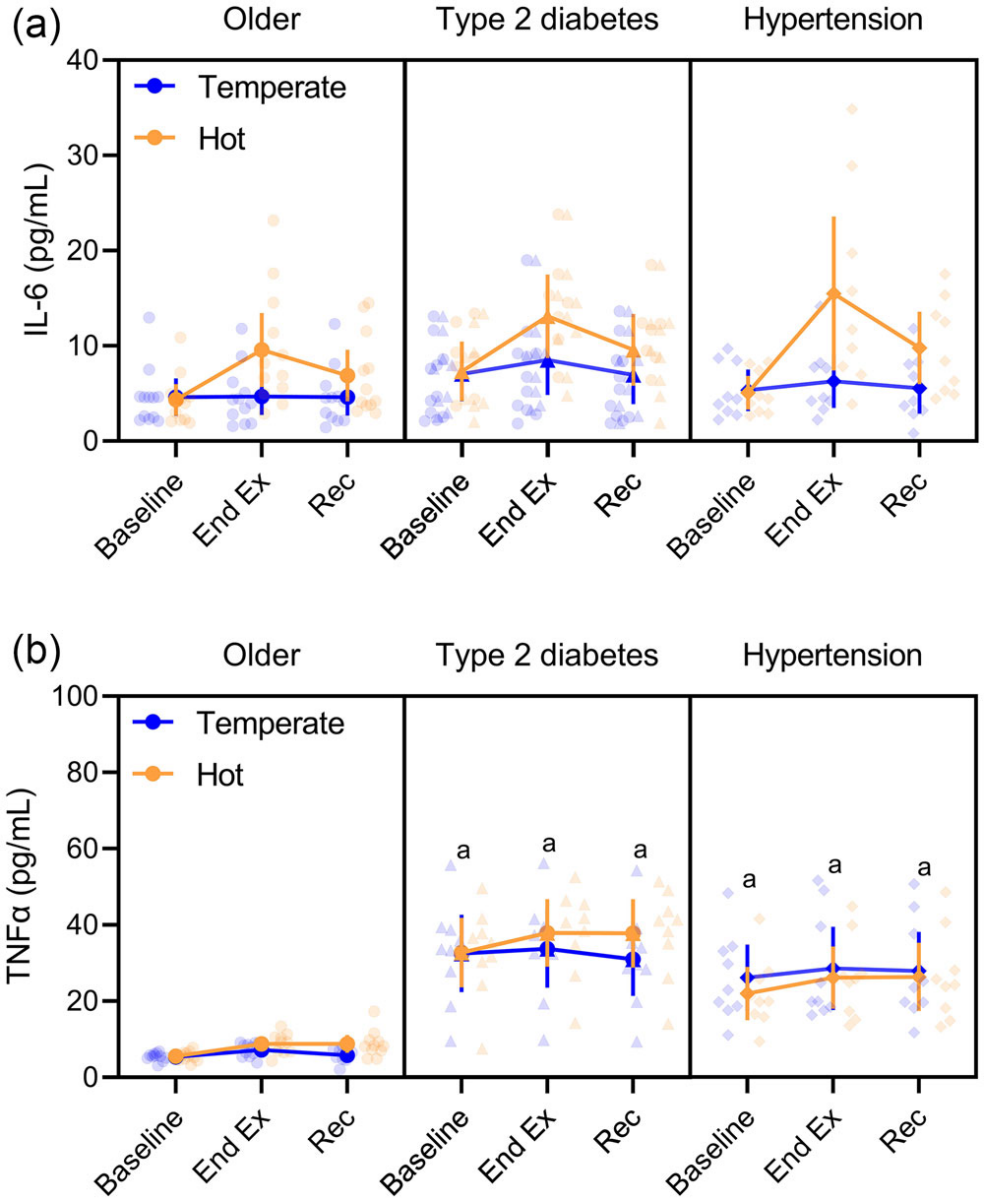
Absolute concentrations for systemic inflammatory cytokines during temperate (blue) and hot (yellow) trials in healthy age‐matched men (*n* = 12; circles), men with type 2 diabetes (*n* = 9; triangles) and men with hypertension (*n* = 9; diamonds). Plasma concentrations of interleukin‐6 (IL‐6; a) and tumour necrosis factor alpha (TNFα; b) are shown before (Baseline) and after exercise (End Ex) and following 60 min of recovery (Rec). Summary data are presented as the geometric mean and geometric SD. Summary data are presented as the mean and SD. Individual data points are shown. Data were compared using mixed‐effects models, with main effects for time and group. ^a^Different from the healthy older group (*p *< 0.05).

In the temperate condition, a main effect of time was observed for IL‐6 (*F* = 6.0, *p *= 0.04). There was no group main effect (*F* = 1.8, *p *= 0.18) or group × time interaction (*F* = 1.78, *p *= 0.15). IL‐6 increased from baseline by 0.83 pg/mL (SD 1.6; *p *= 0.01) but was not different from baseline following recovery (*p *= 1.0; Figure 3a). In the hot condition, there was also a main effect of time for IL‐6 (*F* = 25.0, *p *< 0.001), and no group (*F* = 1.8, *p *= 0.18) or group × time interaction (*F* = 1.3, *p *= 0.28). IL‐6 increased from baseline by 7.15 pg/mL (SD 7.26; *p *< 0.001) and remained elevated by 3.17 pg/mL (SD 4.10; *p *= 0.009) following recovery.

**FIGURE 4:**
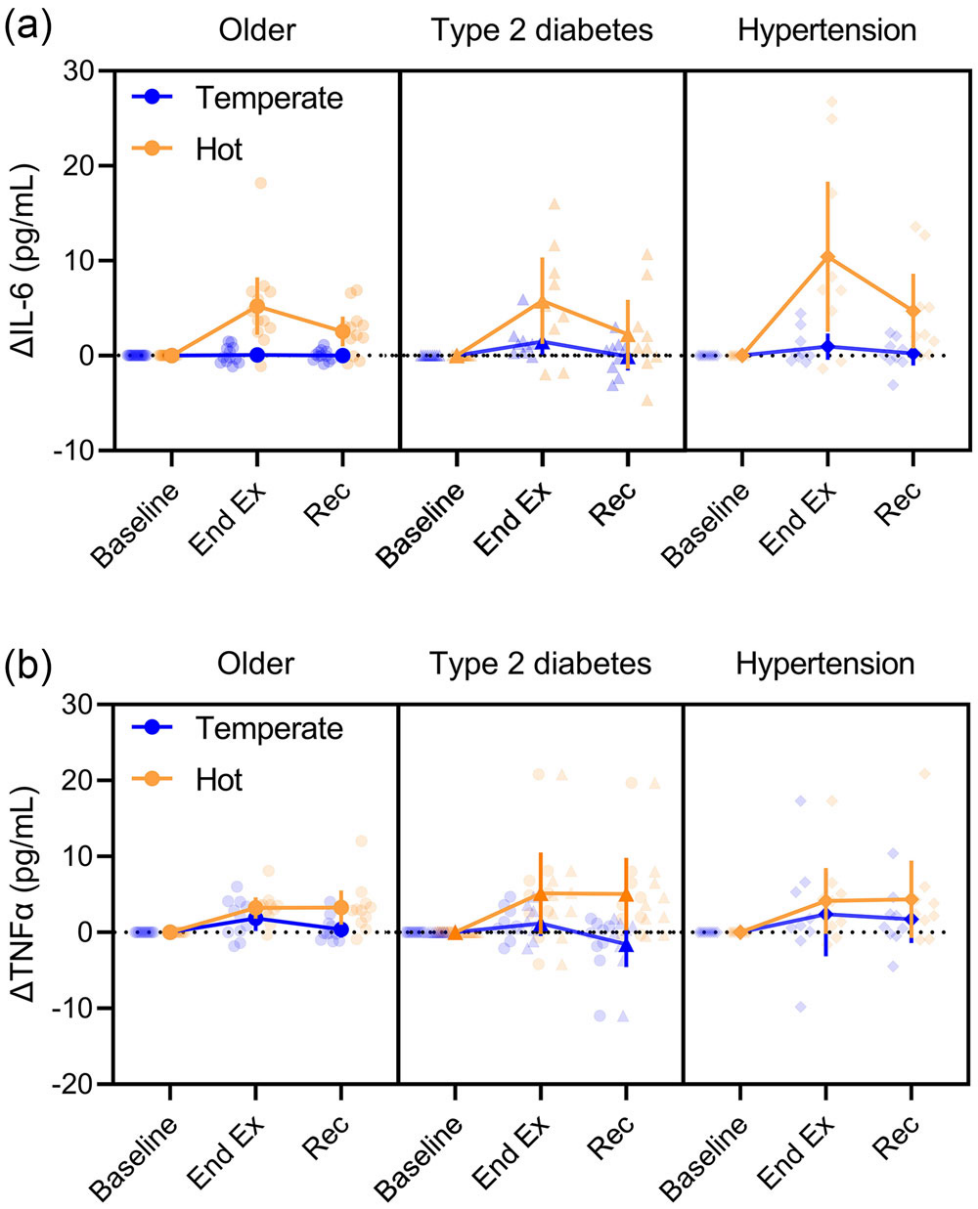
The change in concentrations from baseline for systemic inflammatory cytokines during the temperate (blue) and hot (yellow) trials in healthy age‐matched men (*n* = 12; circles), men with type 2 diabetes (*n* = 9; triangles) and men with hypertension (*n* = 9; diamonds). Plasma concentrations of interleukin‐6 (IL‐6; a) and tumour necrosis factor alpha (TNFα; b) are shown before (Baseline) and after exercise (End Ex) and following 60 min of recovery (Rec). Summary data are presented as the mean and SD. Individual data points are shown.

Relative to the healthy older group, TNFα concentrations were 27.4 pg/mL (19.3, 34.5) greater in the type 2 diabetic group (*p *< 0.001) and 19.3 pg/mL (11.2, 27.4) greater in the hypertensive group (group main effect, *F* = 24.2, *p *< 0.001). TNFα was unchanged between baseline and end‐exercise or between baseline and recovery in the temperate condition (Figure 3b). In the hot condition, TNFα increased by 4.2 pg/mL (SD 5.0; *p *< 0.001) and remained elevated by 4.2 pg/mL (SD 5.3; *p *< 0.01) following recovery. No between‐group differences for the change in TNFα were observed in either condition. The changes in IL‐6 and TNFα from baseline in both the temperate and hot conditions are presented in Figure 4.

## 4 DISCUSSION

To our knowledge, we are the first to report on intestinal epithelial damage, indirect markers of microbial translocation and inflammatory responses during prolonged (180 min), moderate‐intensity exercise with and without heat stress in older men with common chronic diseases (type 2 diabetes or hypertension). In accordance with our prior observations in healthy young and older men (Lee, Flood et al., 2024), we showed small changes in serum IFABP concentrations and no changes in plasma sCD14, plasma LBP or cytokine concentrations in older men with hypertension or type 2 diabetes following prolonged exercise in a temperate environment. In contrast, heat stress led to greater elevations in serum IFABP, plasma sCD14, plasma LBP and IL‐6 when compared with exercise in a temperate environment. Notably, our data indicate that older adults with type 2 diabetes experienced greater enterocyte damage when compared with older adults without chronic health conditions. The hypertensive group experienced similar increases in all proteins when compared with the healthy older group, despite a reduced exercise time and similar end‐exercise body core temperatures, potentially indicating greater heat intolerance and heat vulnerability. Furthermore, LBP, an acute phase reactant synthesized mainly in the liver and considered a surrogate biomarker for the activation of LPS‐induced innate immune responses (Schumann, 1992; Wallett et al., 2021), was greater in type 2 diabetics, and similar in hypertensives relative to the healthy older group following exercise heat stress. Downstream increases in IL‐6 and TNFα were observed in all groups following exercise in the heat, although there were no between‐group differences in these responses. Collectively, our findings extend our prior work by demonstrating greater heat vulnerability in older men with well‐controlled type 2 diabetes or hypertension performing occupational activity in hot environments.

The pathophysiology of heat injury is multifaceted and integrated and is likely to be caused by hyperthermia‐induced reductions in blood flow to the splanchnic viscera to support increased skin perfusion, creating an ischaemic environment, oxidative stress and subsequent gastrointestinal permeability (Meade et al., 2020). Although these haemodynamic adjustments support thermoregulation, the subsequent damage to the gastrointestinal system has been implicated in the pathology of heat stroke (Bouchama et al., 2022; Schlader et al., 2022). Indeed, physiologically relevant increases in body core temperature (i.e., from 37°C to 41°C) disrupt tight junction protein structures, damage intestinal epithelial cells and increase intestinal permeability (Dokladny et al., 2006, 2016). Owing to impairments in the ability to dissipate heat, older adults (>50 years) store 1.3–1.8 times more body heat when exposed to the same heat load relative to young adults (Kenny et al., 2016; Larose et al., 2013; Stapleton et al., 2013). These vulnerabilities are made worse by the presence of common chronic diseases, such as hypertension or type 2 diabetes.

For example, we have recently demonstrated that older men with hypertension or type 2 diabetes have a reduced tolerance to performing prolonged work in hot (32°C wet‐bulb globe temperature) relative to temperate (16°C wet‐bulb globe temperature) conditions, as evidenced by a reduced work time, despite reaching similar cardiovascular and thermal strain compared with their healthy, age‐matched counterparts (Notley et al., 2021). In addition, we have shown that cytoprotective responses to prolonged exercise are altered by environmental heat, occurring earlier in those with hypertension or type 2 diabetes (McCormick et al., 2022). Considering these vulnerabilities, older adults and older adults with common chronic disease states might be at more risk for heat‐related gastrointestinal complications (McKenna et al., 2024). Indeed, our previous work and that of others has shown that older adults, but not younger adults, had increased circulating concentrations of IFABP, a marker of intestinal enterocyte damage, following active heat exposure (Lee, Flood et al., 2024), passive heat exposure (Lee, Russell et al., 2024; Lee et al., 2025) and 3 h of passive heat exposure interspersed with 7.5 min bouts of low‐intensity (3.5 metabolic equivilents) physical activity (Foster et al., 2023). In the present investigation, we demonstrate that type 2 diabetics have a greater end‐exercise increase in IFABP when compared with age‐matched adults without disease. Despite a reduction in work time in individuals with hypertension, we observed similar elevations in IFABP concentrations compared with their healthy counterparts, alongside comparable elevations in both body core temperature and cardiovascular strain. Our observations might indicate that elevated IFABP concentrations are achieved earlier in individuals with hypertension or type 2 diabetes who had reduced work tolerance in the heat. Furthermore, IFABP concentrations remained elevated from baseline in all groups after 60 min of recovery. Although the mechanisms underlying these findings are not known, our data suggest that older adults with type 2 diabetes or hypertension are more susceptible to heat‐induced enterocyte damage during occupational activity. Whether the time course of recovery is different between adults with chronic health conditions and age‐matched control subjects requires further experimentation.

In healthy individuals, the gastrointestinal tract is largely effective in preventing microbial translocation into the systemic circulation (Wells et al., 2017), with the translocation of small amounts of endotoxin occurring during conditions of heat stress (Selkirk et al., 2008). In such conditions, the reticuloendothelial system of the liver provides the first line of gastrointestinal microbial detoxification (e.g., Kupffer cells and hepatocytes) through the portal circulation (Munford, 2005). However, the reticuloendothelial system has a limited capacity for microbial neuralization before leakage into the systemic circulation occurs. Aside from the physiological consequences of exertional heat stress on the gastrointestinal barrier, both ageing and common chronic disease states have also been demonstrated to mediate gastrointestinal barrier permeability (Cox et al., 2014, 2017). For example, with ageing there is an increase in basal inflammatory status, suggested to be caused by prolonged exposure to antigen stress (Müller‐Werdan, 2007) and a decrease in microbial neutralization capacity (Jun Jin et al., 2020). Age‐induced alterations to the gut microbiota have also been associated with an increase in gastrointestinal barrier permeability and cytokine expression (Biagi et al., 2016). In the context of disease state, many studies have demonstrated a link between the gut microbiota and hypertension, with initial research providing evidence that hypertensive patients had increased circulating IFABP, LPS and zonnulin (a precurser protein to haptoglobin, which has a recognized role in disassembling gastrointestinal barrier tight junctions) relative to healthy control subjects (Avery et al., 2021). Furthermore, higher plasma LPS concentrations have been reported in adults with type 2 diabetes compared with a matched control group (Cox et al., 2017).

In the present investigation, we measured sCD14 and LBP, two acute phase proteins involved in the trafficking of LPS to immune cells that can be used as surrogates for LPS translocation (Ogden et al., 2020). LBP is synthesized mainly in the liver, and it binds to LPS and initiates the immune response by presenting LPS to CD14, which, in turn, interacts with Toll‐like receptor‐4 on immune cells (Triantafilou & Triantafilou, 2002). Given that LBP is synthesized and released into the circulation in the presence of LPS and has a relatively long half‐life, the concentration of LBP is considered a surrogate biomarker for activation of LPS‐induced innate immune responses (Guerra Ruiz et al., 2007; Kheirandish‐Gozal et al., 2014; Lepper et al., 2007). In the present study, both LBP and sCD14 were elevated following heat stress, with a greater increase in LBP (but not sCD14) observed in the type 2 diabetics relative to age‐matched control subjects. The increase in LBP might suggest release from intestinal epithelial cells or the liver (Schumann, 1992) and the initiation of the acute phase response. However, any observed LPS translocation did not elicit greater changes in sCD14, which is produced primarily by immune cells (Maliszewski & Wright, 1991), suggesting a comparable immune activation between groups, and could explain why we observed no between‐group differences in IL‐6 and TNFα following exertional heat stress. Despite our observed increases in LBP and the greater increase observed in the type 2 diabetics relative to age‐matched control subjects, LBP concentrations were still generally within the normal range (5–15 µg/mL) in all groups and were substantially lower than peak values observed in traumatic injury (∼200 µg/mL; Cunningham et al., 2006). Taken together, our data suggest that occupational heat stress results in a small amount of microbial translocation and that the degree of microbial translocation might be greater in individuals with type 2 diabetes. However, it should be noted that the increases in downstream inflammatory markers were modest and substantially lower than concentrations seen in individuals suffering from heat stroke (Bouchama et al., 1993, 1991).

### 4.1 Perspectives

Considering the increasing prevalence of hypertension and type 2 diabetes (Kaiser et al., 2018; Mills et al., 2020), in addition to the increase in older adults engaged in physically demanding occupations in a warming climate, it is crucial to improve our understanding of the factors underlying cellular vulnerabilities to heat‐related injuries in occupational settings. Workers are commonly required to perform prescribed work (e.g., a work task at a given pace, intensity and duration), regardless of the climatic conditions, fitness level, age or the presence of common chronic diseases (Kenny, Groeller et al., 2016). Considering that gastrointestinal barrier disruption, microbial translocation and downstream inflammatory processes are thought to play an important mechanistic role in the development of heat‐related injury and exertional heat stroke (Chin & Mackinnon, 2006; Lim, 2018; Lim & Suzuki, 2017), it is important that these responses are understood in older adults and those with chronic conditions. Most studies to date consider healthy young athletic individuals performing sports‐related activities for 1–3 h (Bennett et al., 2020; Etxebarria et al., 2021; Snipe et al., 2017, 2018a, 2018b; Wallett et al., 2021; for a recent meta‐analysis, see Chantler et al., 2021), with few investigations considering the influence of both age and chronic disease on gastrointestinal barrier integrity and surrogate markers of microbial translocation following heat stress (Lee, Flood et al., 2024; Lee, Russell et al., 2024; Lee et al., 2025; McKenna et al., 2024). The present study aimed to improve our understanding of occupational heat stress in heat‐vulnerable older workers. We used a standard heat load to assert the same absolute exercise intensity between participants, more accurately representing day‐to‐day workloads among workers (Meade et al., 2016) and providing the first exploration of the effects of occupational heat stress on enterocyte damage, microbial translocation and inflammation in older individuals with well‐controlled hypertension or type 2 diabetes.

The present study extends our prior observations by demonstrating that older adults with type 2 diabetes undergo greater disruptions to intestinal enterocytes, with concomitantly greater increases in LBP when compared with an age‐matched group without such health conditions. In contrast, there was no difference in IFABP, LBP or inflammatory responses between the hypertensive group and age‐matched controls. When considering similar protein responses despite a reduction in exercise tolerance time in individuals with common chronic disease in comparison to their healthy counterparts, it is possible that individuals with hypertension or type 2 diabetes experienced greater cellular stress relative to healthy older men. This is an important consideration because workers in physically arduous occupations are typically tasked with work shifts that can be 10–12 h in duration, and which are performed over repeated shifts (Kenny et al., 2012; Meade et al., 2015, 2016). Our data support the need for additional controlled studies to further evaluate the effect of occupational thermal stress on the gastrointestinal barrier, microbial translocation and the downstream inflammatory processes that are pathologically linked with heat‐related illness and injury, both acutely, over more prolonged work simulations, and chronically, over repeated work shifts.

### 4.2 Limitations

There are five methodological considerations that require discussion. First, given that this was a secondary analysis of a larger experiment (Notley et al., 2021), we were unable to measure gastrointestinal barrier permeability directly, via a dual absorption sugar test, as is best practice. Instead, we measured IFABP, which is reflective of enterocyte damage, with ourselves (Lee et al., 2024) and others (Foster et al., 2023) observing greater enterocyte damage in older relative to younger participants. More recently, older adults have been demonstrated to be more at risk for gastroduodenal permeability, as evidenced by the greater increases in sucrose excretion in response to 1 h of passive heat stress via a water‐perfused suit (∼2°C change in body core temperature; McKenna et al., 2024). Replication of this work in occupational heat stress models would further delineate the risk posed to older adults. Second, we were unable to measure microbial translocation directly, instead measuring two acute phase proteins (sCD14 and LBP) that are involved in the trafficking of LPS to immune cells and that have been suggested to be surrogates of microbial translocation. Studies incorporating more direct measurements of microbial translocation, which occurs secondary to increased gastrointestinal barrier permeability, such as plasma endotoxin or 16s bacterial ribosomal DNA are warranted (Ogden et al., 2020). Third, women were not included in the present analysis. Although women completed the physiological elements (De Barros et al., 2022a; Notley et al., 2022), complete blood samples were not collected and thus were not included in the present manuscript. Fourth, our findings are restricted to men with either type 2 diabetes or hypertensives without diagnosed neuropathies or other comorbidities and whose conditions were well controlled. Given that the severity of the disease and medications used might independently influence thermoregulatory function, hence the biochemical outcomes, there is a need for larger, confirmatory studies directed at delineating the separate and combined effects of these factors. Fifth, factors other than body core temperature are known to mediate the gastrointestinal barrier response to exercise (e.g., relative work rates and cardiorespiratory fitness; Selkirk et al., 2008; Walter et al., 2021). We recognize that relative exercise intensity plays a role in the biomarkers assessed, and as such, a mechanistic exploration of the factors leading to differential responses in the temperature and hot conditions was not possible. It should be noted that we did not seek to explore mechanisms between the two temperature conditions but to identify whether men with type 2 diabetes have different responses relative to otherwise healthy older adults during exertional heat stress. We incorporated a fixed metabolic heat production model to ensure a matched thermal drive between groups; the type 2 diabetic and hypertensive groups were working at a slightly greater relative intensity in comparison to older adults without chronic disease. The difference in relative work rate might explain some of the variance in serum protein responses in these cohorts. It should be noted that by design, our experimental groups were habitually active and had normal fitness levels for their age, and all individuals with chronic disease enrolled in the present study had controlled health conditions at baseline. Future studies considering the impact of cardiorespiratory fitness and relative work rates on biomarker responses following exertional heat stress in the context of ageing with and without chronic disease are warranted.

## 5 CONCLUSION

We showed that serum IFABP, plasma sCD14 and LBP, surrogate markers of intestinal epithelial injury, immune activation and LPS exposure, are unaffected following prolonged exercise in a temperate environment in older men with well‐controlled type 2 diabetes or hypertension. However, when exercise is performed in the heat, we observed greater elevations in IFABP in older men with type 2 diabetes, occurring despite disease‐associated reductions in exercise tolerance time. Similar end‐exercise changes in cytokines were observed in all groups following exercise in the heat. Collectively, these results suggest that when compounded by type 2 diabetes or hypertension, older workers might undergo greater gastrointestinal barrier disruption during prolonged physical work in the heat, underlying a greater vulnerability to downstream (e.g., exertional heat illness, systemic inflammation) heat‐induced injury.

## AUTHOR CONTRIBUTIONS

Glen P. Kenny, Ben J. Lee and James J. McCormick conceptualized and designed the research. Kelli E. King, James J. McCormick and Sean Notley performed data collection. Ben J. Lee, Tessa R. Flood and Sophie L. Russell performed the blood analysis. Ben J. Lee, Tessa R. Flood and Sophie L. Russell performed statistical analysis and prepared figures. Ben J. Lee drafted the manuscript. All authors interpreted the results. All authors edited and revised the manuscript, approved the final version of the manuscript and agree to be accountable for all aspects of the work in ensuring that questions related to the accuracy or integrity of any part of the work are appropriately investigated and resolved. All persons designated as authors qualify for authorship, and all those who qualify for authorship are listed.

## CONFLICT OF INTEREST

None declared.

## Data Availability

Individual data are presented within this manuscript (Figures 1, 2, 3, 4). The data that support the findings of this study are available from the corresponding author upon reasonable request.
